# Evaluating viral interference between Influenza virus and Newcastle disease virus using real-time reverse transcription–polymerase chain reaction in chicken eggs

**DOI:** 10.1186/1743-422X-9-128

**Published:** 2012-07-01

**Authors:** Shengqiang Ge, Dongxia Zheng, Yunling Zhao, Hualei Liu, Wenbo Liu, Qing Sun, Jinming Li, Songmei Yu, Yuanyuan Zuo, Xiuju Han, Lin Li, Yan Lv, Yingli Wang, Xiufan Liu, Zhiliang Wang

**Affiliations:** 1 Animal Infectious Disease Laboratory, College of Veterinary Medicine, Yangzhou University, Yangzhou, 225009, China; 2 China Animal Health and Epidemiology Center, Qingdao, Shandong, 266032, China

**Keywords:** Viral interference, Influenza virus, Newcastle disease virus, Real-time RT-PCR

## Abstract

**Background:**

Simultaneous and sequential allantoic cavity inoculations of Specific-pathogen-free (SPF) chicken eggs with Influenza virus (AIV) and Newcastle disease virus (NDV) demonstrated that the interaction of AIV and NDV during co-infection was variable. Our research revisited the replication interference potential of AIV and NDV using real-time reverse transcription–polymerase chain reaction (real-time RT-PCR) for AIV and NDV to specifically detect the viral genomes in mixed infections.

**Results:**

Data from this survey showed that when different doses of NDV (Lasota or F48E8) and AIV (F98 or H5N1) were simultaneously inoculated into embryonating chicken eggs (ECE), interference with the growth of NDV occurred, while interference with the growth of AIV did not occur. When equal amount of the two viruses were sequentially employed, the degree of interference was dependent upon the time of superinfection and the virulence of virus.

**Conclusion:**

AIV have a negative impact on NDV growth if they are inoculated simultaneously or sequentially and that the degree of interference depended upon the quantity and relative virulence of the virus strains used; however, interference with AIV was not observed. Only if NDV were inoculated at an earlier time will NDV able to interfere with the growth of AIV.

## Background

Avian influenza (AI) and Newcastle disease (ND) are the most devastating diseases of poultry and other avian species recognized worldwide. Both AI and ND are RNA-viral diseases of birds, caused by type A orthomyxoviruses and type 1 avian paramyxoviruses respectively, with several traits in common. They are major concerns of animal husbandry, due to severe economic losses to the poultry industry and fulminating infection accompanied by high mortality [1]. In addition, after disease outbreaks, trade restrictions and embargoes may also be imposed by other countries.

Understandably, both diseases were added to list A within the category of avian diseases by the Office International des Epizooties (OIE), the official international organization for animal health and sanitary standards under the World Trade Organization. In the case of AI, it is not only of importance in causing illness and death to chickens, but also as a threat to human health.

Mixed infections of avian species with NDV and AIV have been reported before [2]. However, multiple infections between AIV and NDV were rarely observed [3]. Recently, it has been proven that if NDV and AIV are both present in a cloacal sample, the advantageous growth of NDV may inhibit AIV propagation yielding false-negative AIV results [4]. This could be explained by a hypothesis that interference may exist between AIV and NDV. Since 1935, when the first study on interference between animal viral infections was described by Hoskins [5], viral interference has been studied extensively in a wide variety of animal and plant viruses [6,7]. The number and diversity of these experiments to date vividly witness the fact that mixed infections of animal and plant viruses are a common phenomenon [8-13]. However, although AIV and NDV are causative agents of the most important diseases of aves, there are, to our knowledge, only six reported studies on interference between AIV and NDV. The first study was performed by Burnet [14]. He presented a linear arrangement of these two viruses and found that cells treated with any one of them were subsequently refractory to agglutination by the other virus. Shortly after, Florman, A. L proved that after 24 hours chick embryos infected by NDV are no longer susceptible to infection even with high level doses of the PR8 strain of influenza A virus [15]. Subsequently, Bang, F.B [16] found that small doses of Newcastle virus failed to produce the usual lethal effect if the chick embryos had been previously infected with the influenza virus. With further investigation, Carr, J. H demonstrated that the more important factor in viral interference between swine influenza virus(SIV) and NDV is the inoculation differential [17]. A fifth paper was published by Kennedy F. Shortridge and Alison P. King in 1983 [3]. They found that when NDV (avian PMV-1) was co-cultivated with AIV, AIV was preferentially detected over the NDV in allantoic fluid. The last study was performed by Wenbo Liu et al [18]. Their results showed that H9 subtype AIV causes strong interference in the propagation of NDV in chicken embryos. However, none of these studies have quantitatively evaluated the degree of interference between these two viruses. The chief shortcoming of former studies of interference is the relative lack of differentiating technology to distinguish individual viruses and the inability to determine the exact number of virus particles that participate in production of progeny. Owing to the important position of these two viruses, therefore, it is of theoretical and practical importance to continue discussions about viral interference by evaluating AIV and NDV replication using a new detection technique, real-time RT-PCR.

## Results

### Standard curve and sensitivity of real-time RT-PCR

Standard curve was generated from the amplification of 10-fold diluted (1×10^7^~1copies/μL) in vitro-transcribed RNA in the Real-time RT-PCR assay and analyzed using the 7500 System SDS Software Version 1.2.2. Threshold cycle (Ct) values represented the cycle number at which the significantly increased fluorescence was first detected or the crossing point at which the amplification curve exceeded the threshold line. The consistency of replicates was measured by the correlation coefficient (R2), which indicates the linearity of Ct values plotted in the standard curves and that was obtained from linear regression analysis. The R2 index for AIV and NDV genes was 0.998 and 0.997, respectively. As the slope of the standard curves of AIV and NDV were 3.046 and 3.220 respectively, the efficiency of the reaction defined by 10(−1/slope) were 2.13 and 2.04, which were within the acceptable range of 1.7 to 2.2 [19]. According to the standard curve, the linear equation for the real-time RT-PCR for AIV and NDV was y = −3.046x + 37.3352 and y = −3.22x + 35.9949, respectively. The sensitivity of this method defined by the lowest concentration of the AIV in one PCR assay was 10copies/reaction(approximately 10^0.790^ EID_50_ for F98, 10^0.480^ EID_50_ for H5N1), the same sensitivity as for the NDV(approximately 10^1.002^ EID_50_ for Lasota, 10^1.040^ EID_50_ For F48E8).

### Observed products of co-cultivation

**Figure 1:**
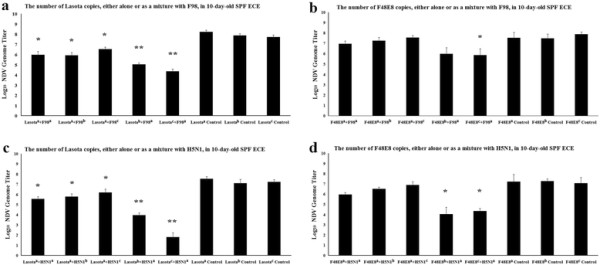
**The replication of NDV (Lasota of F48E8) in ECE after infection alone or as a mixture with AIV (F98 of H5N1) were measured by using the Real-time PCR.** Supersript a represents an input multiplcitu pf 10^3^ EID_50_ per chicken egg. Superscript b represents an input multiplicity of 10^2^ EID_50_ per chicken egg. Superscript c represents an input multiplicity of 10^1^ EID_50_ per chicken egg. Bars represents the standard deviations for two experiments carried out on three eggs. Statistical significance between the experimental group and control group was determined by the student’s t-test. *p <0.05, **p < 0.01,***p < 0.001.

**Figure 2:**
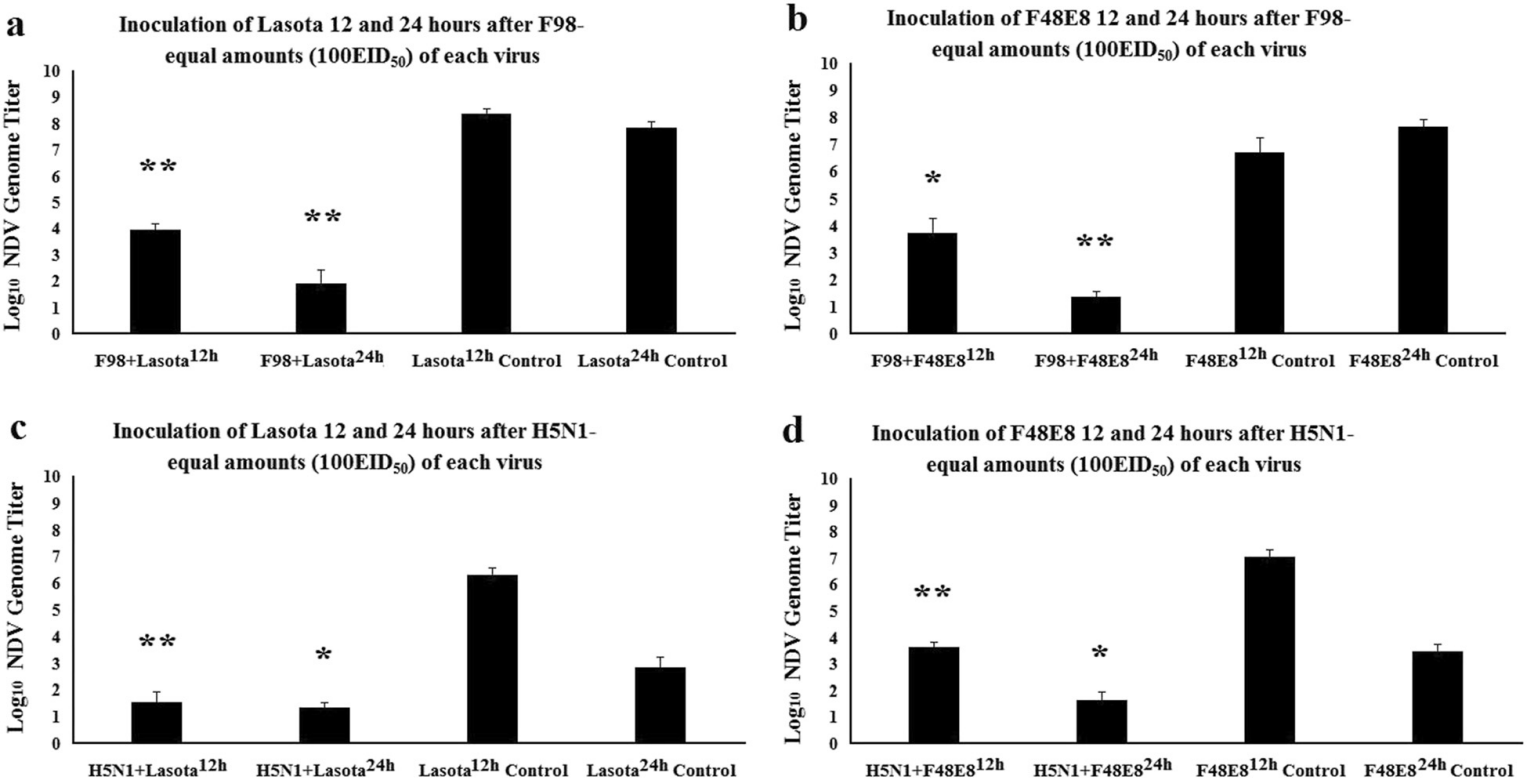
**The replications of NDV (Lasota of F48E8) in ECE after inoculated first with AIV (F98 of H5NI) were measured by using the Real-time PVR.** Superscript 12 h represtnets 12 hours interval between two viruses. Superscripts 24 h represents 24 hours interval between two viruses. Bars represent the standard deviations for two experiments carried out on three eggs. Statistical significance between the experimental group and control group was determined by the student’s t-test. *p<0.05, **p<0.01, ***p<0.001.

**Figure 3:**
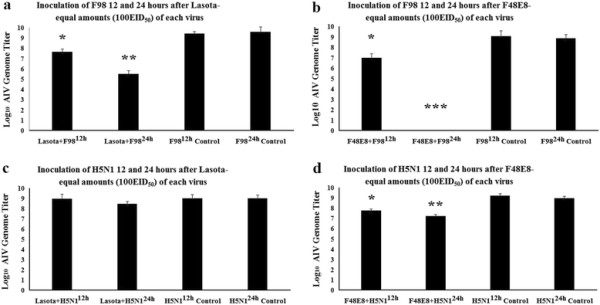
**The replications of AIV (F98 or H5N1) in ECE after inoculated first with NDV (Lasota or F48E8) were measured by using the Real-time PCR.** Supercript 12 h represtents 12 hours interval between two viruses. Superscript 24 hours interval between two viruses. Bars represents the standard deviations for two experiments carried out on three eggs. Statistical significance between the experimental group and control group was determined by the student’s t-test. *p<0.05, **p<0.01, ***p<0.001.

The aim of the present study was to obtain information on quantitative and temporal factors that affect interference between AIV and NDV. Two experiments were done to determine whether co-infections of ECE by F98 or H5N1 (AIV) and Lasota or F48E8 (NDV) exhibited an interference interaction. By using real-time RT-PCR, the interference level between them can be quantitatively evaluated. The results of experiment 1 are shown in Figure 1; results of experiment 2 are shown in Figure 2 and 3.

The purpose of the first set of experiments are to determine whether there was a pattern of interference related to the absolute amount of virus particles or a particular ratio of AIV to NDV. The replications of AIV or NDV in ECE after infection alone or as a mixture consisting of different EID_50_ of each virus were measured. Based on real-time RT-PCR assays, replication interference by AIV strains H5N1 and F98 on NDV strain Lasota was very apparent (Figure 1a, c). Virus yield of Lasota from dually infected ECE with H5N1 and F98 were significantly lower than those from singly infected ECE. It was shown that the higher the level of infection with AIV virus, the greater the degree of interference occurred. However, a striking difference was found between the avirulent strains and virulent strains of NDV, in as much as virulent F48E8 was more powerful than avirulent Lasota in resisting interference induced by AIV. When equal multiplicities of the AIV (H5N1 or F98) and F48E8 were employed, only slight signs of interference on F48E8 were observed (Figure 1b, d). Only high amounts of AIV can cause interference with F48E8 (P<0.05). Although no significant differences in F48E8 was detected between single and doubly infected ECE when virulent F48E8 was inoculated with equal or lower amount of the AIV(H5N1 and F98), it was still apparent that the amount of F48E8 in doubly infected ECE was relatively lower (Figure 1b, d). Another finding was that virulent H5N1 was a more powerful agent than avirulent F98 in preventing the multiplication of F48E8. When F48E8 was co-inoculated with 10 times more H5N1, a significant difference in F48E8 could occurred (Figure 1d), whereas for F98, 100 times more were required to affect a significant difference (Figure 1b). In view of the interfering effect of NDV (Lasota and F48E8) on AIV, NDV failed to inhibit the growth of AIV.

In the second set of experiments, the changes in interference level when time is allowed to elapse between inoculation by AIV and NDV were measured. When AIV and NDV were inoculated at different time intervals, interference phenomenon was somewhat different from simultaneous inoculation. There was a strong inhibition of the growth of NDV when AIV was inoculated 12 hours earlier than NDV (Figure 2a-d). The degree of interference was considered to be significant for values of P<0.01 expect the inoculation of F48E8 12 hours after F98 (p<0.05) (Figure 2b). It is noteworthy that when H5N1 was inoculated 12 hours earlier than NDV, the result of interference level was significant at P<0.01, whereas when H5N1 was inoculated 24 hours earlier than NDV, the result was at P>0.05 (Figure 2c, d). It is probably due to the fact that less than 24 hours was left for replication of the NDV control because most of the death of ECE inoculated with H5N1 occurred between the 36th and 48th hour after inoculation. That is also explained that LaSota/24 h and F48E8/24 h control NDV had different titers (in Figure 2a vs. Figure 2c and Figure 2b vs. Figure 2d, respectively) because LaSota/24 h and F48E8/24 h control NDV in Figure 2a and Figure 2b has longer culture time compare to LaSota/24 h and F48E8/24 h control NDV in Figure 2c and Figure 2d. When the NDV was inoculated earlier than AIV, the interference on AIV by NDV was more complicated. ECE inoculated first with Lasota and re-inoculated 12 and 24 hours later with F98 resulted in fewer amounts of F98 (Figure 3a). The degree of interference increased with the time that elapsed before superinfection with the AIV. This is similar to the result of F48E8 with H5N1 (Figure 3d). However, it was clearly shown that Lasota was not capable of interrupting the multiplication of H5N1 even when introduced as early as 24 hours before H5N1 (Figure 3c). Maximal inhibition of the growth of AIV was observed when F48E8 was inoculated 24 hours earlier than F98 (Figure 3b). None of the AIV genome was detected in this situation, showing that F48E8 was able to suppress completely, (or almost completely) the growth of F98 in ECE inoculated with F48E8 24 hours before. The stronger capacity of F48E8, compared with Lasota, in preventing the multiplication of AIV, (inoculated later) corresponds to the greater interfering capacity of F48E8 over that of Lasota, which was clearly demonstrated in this study. It seems reasonable to conclude that the virulence of a virus and its interfering capacity are closely related.

## Discussion

Interference of AIV (H5N1 and F98) with NDV (Lasota and F48E8), and vice versa, was studied in the ECE system for simultaneous and relatively short intervals between inoculations. In this system, a number of variables have been measured, e.g. the effects of varying the dose of interfering and challenge virus or the time interval between the two inoculations and the use of different virus strains. We report here for the first time an evaluation of the interference between AIV and NDV in terms of viral replication using real-time RT-PCR. The results of the experiment clearly show that AIV inhibited the growth of NDV, and that the degree of interference depended upon the quantity and relative virulence of the virus strains used. The studies also indicate that NDV can’t suppress the growth of AIV even when the NDV was given a growth advantage in the ECE by being inoculated at a higher EID_50_ than AIV. With the virus doses and intervals between inoculations described, significant reduction of the genome titer of the AIV only occurred when NDV was inoculated prior to AIV. However, although infection of ECE by AIV uniformly interfered with subsequent infection by NDV, a primary infection by NDV was not always able to prevent later infection by AIV. For instant, even when Lasota was inoculated 24 hours prior to H5N1, there was still no interference on H5N1 (Figure 3c). Therefore, it seems clear that Lasota did not induce interference against H5N1 multiplication either with simultaneous or preoculation with Lasota.

Comparison of the results obtained from this study pointed to a similar qualitative conclusion with other studies, viz. that AIV was found to be a far more powerful agent than NDV in causing interference between these viruses. Additionally, the virulence of the virus strains was another important factor that affects interference in the reciprocal interference. In other words, the virulent strain of NDV is more puissant than avirulent strain in resistanting interference induced by AIV. It can be explained by the theory that the NDV replication is directly associated with the virulence of virus [20]. The above findings in general agree with those reported by Shortridge and King, who explored co-cultivation of AIV and NDV in ECE [3], and also by Wenbo Liu et al., who studied H9-NDV interference in chicken embryo [18]. However, a controversial finding was reported by Zowalaty et al that in the presence of mixed infection with NDV and AIV in cloacal swabs, NDV could be recovered from ECE but not AIV [4]. This different discovery may be due to the presence of inactivated AIV or large quantitative difference between AIV and NDV in cloacal swabs. In our study, the NDV was added into ECE at an input multiplicity of 1000 EID_50_ per chicken egg, whereas the AIV was used at a multiplicity of 1000,100 and 10 EID_50_ per chicken egg. If the AIV was used at a multiplicity of 1 EID_50_ or less, we may find the similar result as describe by Zowalaty.

Interference could have taken place either on or within the available cells. Different mechanisms have been proposed to explain viral interference. These have been classified broadly into two categories: (i) attachment interference, and (ii) intracellular interference. The former is mediated through blockade or destruction of available receptor sites for the superinfecting virus. The latter involves virus-induced interferon interference, or competition for replication sites or essential factors of viral replication, or formation of defective interfering (DI) particles, etc. [21].

Virologist’s enormous interest in the observation of viral interference phenomena led to the discovery of interferon. In the early 1940s, Henle & Henle [22] and Ziegler & Horsfall [23] discovered that an inactivated influenza virus particle was capable of interfering with the multiplication of live virus added later. This finding opens the door to study viral interference mediated by the interferon system. Subsequent intensive research proved that interferon production was a common event and it inhibits the growth of many viruses, certainly including AIV and NDV [24]. Other experiments additionally found that in order to establish interferon-mediated interference, several hours was required to create interferon [25]. AIV and NDV are capable interferon inducers and their ability to induce the formation of interferon is closely related with their virulence [26]. The basic characteristics of interferon-mediated interference mentioned above agree with the observation of experiment 2 in this study. That is, the pre-inoculated virus always inhibited the growth of superinfection virus. However, in experiment 2, an exception occurred in the Lasota inoculation prior to inoculation with H5N1. This is probably due to the fact that Lasota is a weak interferon-inducer. 24 hours is not sufficient time for Lasota to produce inhibition-level interferon to suppress the growth of H5N1. This fits precisely with one of the properties of interferon, viz that the time of induction of maximum interferon depends on the virulence of the strain [26]. However, for experiment 1 in this research, when AIV and NDV inoculations were made at the same time, interference always occurred in NDV. This phenomenon is not easily explained by interferon-mediated interference because, according to the literature, in order to establish interferon-mediated interference, several hours are required to establish interference [27]. That is also the reason why most virus interference phenomena were not interpreted by physical blockade of receptors.

A simple but likely explanation for the interference between AIV and NDV in experiment 1 could be the competition for the same receptor. It has been clearly established that the cell surface receptor for AIV is sialic acid-containing glycoconjugates [28], whereas the cellular receptors for Newcastle disease virus have been proposed as Gangliosides and N-glycoproteins, both of which contain sialic acid [29]. These findings imply the existence of a common receptor site on permissive host cells shared by AIV and NDV [30], and raise the possibility that when these two viruses are inoculated into the same egg, they could compete for shared virus receptor-sialic acid which is essential for virus adherence. Homologous and heterologous viral interference induced by blocking or destruction of viral receptors among NDV or AIV has long been described [31,32]. Therefore, theoretically, when AIV and NDV were simultaneously inoculated into the ECE, there was obvious interference, probably due to direct competition for the same viral receptors on the cell surfaces.

In addition, AIV and NDV are both (−)sense RNA viruses, grouped under the term myxovirus, due largely to their property to adsorb onto the erythrocytes cell-surface receptors of fowl, causing their agglutination, a striking biologic property common to all of myxovirus [33]. Also to be considered is that AIV and NDV do exhibit certain similarities. In aves, the clinical symptoms of highly pathogenic avian influenza A (HPAI) are very similar to those of a severe attack of virulent NDV strain.

## Conclusions

AIV have a negative impact on NDV growth if they are inoculated simultaneously or sequentially and that the degree of interference depended upon the quantity and relative virulence of the virus strains used; however, interference with AIV was not observed. Only if NDV were inoculated at an earlier time will NDV able to interfere with the growth of AIV.

## Methods

### Specific-pathogen-free (SPF) chicken eggs

Fertile eggs were purchased from SPF chicken Research Center of Shandong Institute of Poultry Science (Jinan, China). The eggs were incubated for 10 days, and then used for virus isolation attempts after challenge with NDV and AIV. SPF chicken eggs were used in confirmatory experiments only.

### Virus strains

The virus strains employed in this study included avirulent and virulent strain of AIV and NDV. Lasota (Chinese reference avirulent NDV strains of chicken) and F48E8 (Chinese reference virulent NDV strains of chicken) were obtained from National Reference Laboratory for Newcastle Disease. Reference avirulent strain of A/Chicken/Shanghai/F/98(F98, H9N2) was kindly provided by College of Veterinary Medicine, Yangzhou University. The separate strain of H5N1 was obtained from China Animal Health and Epidemiology Center, Qingdao. These four viruses are maintained as low embryo passage viruses in our laboratory. Before each experiment, the hemagglutination titer (HA) and fifty-percent infectious dose (EID_50_) titers of each virus stock were determined. EID_50_ titers of these four viruses were determined by serial titration in 10-day-old SPF ECE and calculated by the method of Reed-Muench. All experiments with infectious H5N1 virus were conducted under BSL-3 containment.

### Virus isolation and RNA extraction

All virus isolates were grown and passaged in 10-day-old SPF ECE. Allantoic fluids with viruses were collected and RNA extraction was carried out with the High Pure Viral RNA Kit (Roche Applied Science, Mannheim, Germany) according to the manufacturer’s protocol.

### Primer and probe design

The nucleotide sequences of the matrix (M), conserved for Influenza A and Newcastle disease virus, was selected for detection of type A influenza virus and Newcastle disease virus. The M gene-specific primer and probe set for type A influenza virus was cited from those described previously [34]. The M gene-specific primer and probe set for Newcastle disease virus was modified from those described previously [35]. The oligonucleotide sequences for the primers and probes were as follows

AIV M TaqMan® Forward, 5′-AGGTCGAAACGTAYGTTCTCTCTAT-3′;

AIV M TaqMan® Reverse, 5′-GGTCTTGTCTTTAGCCAYTCCAT-3′;

AIV M Probe, 5′-[JOE]-TCAGGCCCCCTCAAAGCCGA-[TAMRA]-3′;

NDV M TaqMan® Forward, 5′-AGTGATGTGCTCGGACCTTC-3′;

NDV M TaqMan® Reverse, 5′-CCTGAGGAGAGGCATTTGCTA-3′ and

NDV M Probe, 5′-[FAM]-TTCTTCTCTGGCAGTGGGACAGC-[TAMRA]-3′.

All the primer and probe sets were designed and analyzed by using software Primer Premier (Version5.0; PREMIER Biosoft International, CA).

### Real-time RT-PCR analyses

The real-time RT-PCR was carried out using the commercial One step PrimeScript RT-PCR Kit (TaKaRa Biotechnology Co., Dalian, China) in a 20 μL reaction mixture containing 10 μL of 2×One step RT-PCR Buffer III, 0.4 μL TaKaRa Ex Taq Hs(5U/μL), 0.4 μL PrimeScript RT Enzyme MixII, 0.4 μL ROX Reference DyeII(50×), 5.2 μL RNase free dH_2_0, additional 2 μL RNA sample, and 0.8 μL of primers and probes respectively. Each primer and probes for NDV and AIV were used at a final concentration of 10 μM. The real-time reaction was conducted on the 7500 real-time PCR System (Applied Biosystems by Life Technologies, Foster City, CA). The fluorescence RT-PCR program consisted of 5 min at 42°C for reverse transcription, 10 s at 95°C for activation of Taq enzyme, followed by 40 cycles for amplification with 95°C for 5 s, 58°C for 1 min and a final extension at 72°C for 34 s. The fluorescence signals of FAM and JOE were acquired at the end of each elongation step. For each dye, the analysis of fluorescence data was conducted using the SDS software (Version 1.4; Applied Biosystems by Life Technologies, Foster City, CA). The threshold fluorescence level, used to derive Ct values, was determined automatically by the SDS software.

### The limit of detection (LoD) of the real-time RT-PCR assay

In vitro-transcribed RNAs of the NDV and AIV genes were analyzed to determine the LoD of the assay in terms of RNA copy numbers. For the M gene primer-probes of AIV and NDV, Lasota and F98 cDNA were amplified with their M gene-specific primer. PCR products were then cloned into pGEM-T Easy Vector (Promega Corporation, Madison, WI) and linearized by endonuclease digestion at a unique site (SalI). In vitro-transcribed RNA was generated from the T7 promoter following the manufacturer’s recommendation (TranscriptAid™ T7 High Yield Transcription Kit, Fermentas International Inc, Harrington Court, Burlington). RNA was quantified spectrophotometrically following enzymatic removal of DNA. The number of RNA copies was calculated by following the formula reported in a previous study [36]. Tenfold dilution of the RNA transcripts, ranging from 1 to 10^7^ copies/μL, were prepared. The LoD of the assay was determined from three independent replicates.

### Experimental designs for the reciprocal interference studies between AIV and NDV

The interference phenomenon was studied by two experiments, described below:

Experiment 1: SPF 10-day-old ECE were simultaneously infected with two viruses at different multiplicities; the AIV or NDV was added at an input multiplicity of 1000 EID50 per chicken egg, whereas the challenge virus was used at a multiplicity of 1000,100 and 10 EID50 per chicken egg. An equally large group of ECE received AIV or NDV alone as controls; in these, sterile phosphate buffered saline (PBS) was injected instead of another virus. After injection, ECE were candled at 12 hour intervals. Embryos dead at the first candling were discarded. At the succeeding candlings, allantoic fluids from eggs with dead embryos and the corresponding control group were harvested at the same time. However, the avirulent strain Lasota and F98 aren’t lethal for ECE, so their allantoic fluid was harvested at the time of 96 hours after inoculation. All the experiments were repeated twice. The genome titer of each virus was determined using the Real-time PCR. The degree of interference was estimated by comparing AIV or NDV yields from dually (AIV and NDV) infected ECE with those of the corresponding controls as measured independently by real-time RT-PCR. Statistical significance between the experimental group and control group was determined by the Student’s t-Tests.

Experiment 2: This experiment was set up to investigate the effect of first-inoculation virus on the growth yield of second-inoculation virus in the tissues of the ECE. Experiments were designed in such a way, that, when the second inoculation was performed, all ECE were already infected with another virus introduced as the first agent. ECE were infected with AIV or NDV with 100EID50, 0.1 ml per egg. The same amount of control eggs were inoculated with Sterile PBS. After incubating for 12 or 24 hours and candling for viability, the living eggs, including both the experiment group and control group, were re-inoculated with another virus into the allantoic cavity. By the second day after inoculation with virulent strain of F48E8 and H5N1, nine tenths of the embryos were dead. Allantoic fluids were harvested from three dead eggs of the groups receiving the F48E8 or H5N1. As controls, similar harvests were made from eggs which had been inoculated with Sterile PBS followed by the virulent virus. In the groups inoculated first with avirulent strains of Lasota or F98 and re-inoculated with F98 or Lasota, ECE can’t die, so the harvests were collected from three live eggs from the experiment group and control group at the time of 96 hours after first inoculation. After harvesting, the genome titer of each virus and the extent of interference were determined as described above.

## Competing interests

This work was funded by Major State Basic Research Development Program of China (973 Program)(grant number 2011CB505003).The Earmarked Fund For Modern Agro-industry Technology Research System (nycytx-41-G07) and supported in part by the grants from the National Natural Science Foundation of China (30901079).

## Authors’ contributions

ZW created the original idea of this research. ZW and SG designed the whole experiments and conducted analysis and interpretation of the data. SG, DZ, YZ and HL carried out the most of experiments and drafted the manuscript. WL, QS, JL, SY, YZ, XH, LL, YL, YW and XL participated in partial research work. All authors read and approved the final manuscript.
